# Regulation of C-Type Lectin Receptor-Mediated Antifungal Immunity

**DOI:** 10.3389/fimmu.2018.00123

**Published:** 2018-02-01

**Authors:** Juan Tang, Guoxin Lin, Wallace Y. Langdon, Lijian Tao, Jian Zhang

**Affiliations:** ^1^Department of Nephrology, Xiangya Hospital, Central South University, Changsha, Hunan, China; ^2^Department of Pathology, The University of Iowa, Iowa City, IA, United States; ^3^Department of Anesthesiology, The Third Xiangya Hospital, Central South University, Changsha, Hunan, China; ^4^School of Biological Sciences, University of Western Australia, Perth, WA, Australia

**Keywords:** C-type lectin receptor, fungal infection, signaling pathways, posttranslational modifications, immunity

## Abstract

Of all the pathogen recognition receptor families, C-type lectin receptor (CLR)-induced intracellular signal cascades are indispensable for the initiation and regulation of antifungal immunity. Ongoing experiments over the last decade have elicited diverse CLR functions and novel regulatory mechanisms of CLR-mediated-signaling pathways. In this review, we highlight novel insights in antifungal innate and adaptive-protective immunity mediated by CLRs and discuss the potential therapeutic strategies against fungal infection based on targeting the mediators in the host immune system.

## Introduction

Fungi are ubiquitously present in the mucosal and epidermal surfaces in healthy individuals and often cause infections in immune-compromised patients. These include HIV-positive patients, recipients of organ transplants, and cancer patients treated with chemotherapy. In healthy individuals, fungal infections can also develop including vulvovaginal candidiasis, tinea pedis, fungal keratitis, and chromoblastomycosis (1–6). Invasive fungal infections, particularly with *Candida albicans* (*C. albicans*), demonstrate high mortality rates and kill more than 1.5 million people worldwide annually (7). Moreover, other identified pathogenic fungi such as *Aspergillus fumigatus* (*A. fumigatus*), *C. auris*, and *Cryptococcus gattii* (*C. gattii)* also pose a great threat to public health (1, 8, 9). Toxicity and resistance to the limited number of antifungal agents that are currently available contributes to high morbidity and mortality associated with fungal sepsis. Therefore, there is an urgent need to better understand the immune response during fungal infection and develop new immuno-therapeutic approaches.

C-type lectin receptors (CLRs), including transmembrane and soluble forms, are characterized by containing at least one C-type lectin-like domain (CTLD). They have been shown to recognize both endogenous and exogenous ligands (10). As the most important pattern recognition receptor (PRR) family for the detection of fungi, CLRs are recognized to play a critical role in tailoring immune responses against fungal exposure (11–14). In this review, we will focus on the roles and mechanisms of membrane-bound CLR-mediated-signaling pathways in host defense against fungal infections, with an emphasis on *C. albicans*. *C. albicans* is the most common fungal species isolated from biofilms, formed either on implanted devices or on human tissues, which become pathogenic in immune-compromised patients. We will also discuss the role of posttranslational modifications (PTMs) of CLR-signaling pathway components in anti-fungal immunity. In addition, we will also summarize the recent progress on the potential host-derived immune therapies for disseminated candidiasis.

## Fungal Recognition

The pathogen-associated molecular patterns (PAMPs) on the fungal cell wall are crucial for the initiation of innate immune responses against fungal pathogens. The fungal cell wall is predominantly composed of carbohydrate polymers interspersed with glycoproteins (15–17). The three major components, found in almost all fungi, are β-glucans, which are anchored in the inner core of the cell wall, chitin, which is a robust β-1,4-linked homopolymer of N-acetylglucosamine (GlcNac) located in the inner cell wall, and mannans, which are localized in the outer layer of the fungal yeast cell wall. The central core of the cell wall is branched β-1,3/1,6-glucans that are linked to chitin *via* β-1,4 linkages (15, 18). Mannans are chains of up to several hundred mannoses that are added to fungal proteins *via* N- or O-linkages. Mannoproteins can covalently attach to glucans or chitin *via* either their sugar residues or glycosylphosphatidylinositol (17). In addition, O-linked glycoproteins containing mannobiose-rich structures from *Malassezia* function as distinct ligands to induce immune responses (19). Another crucial component on the fungal cell wall is melanin, which is involved in fungal virulence, resistance to antifungal drugs, and protection against insults from the environment (20, 21).

The recognition of PAMPs expressed in pathogens involves four families of PRRs, including Toll-like receptors (TLRs), NOD-like receptors (NLRs), retinoic acid-inducible gene I (RIG-I)-like receptors (RLRs), and CLRs, each of which shows notable differences regarding pathogen recognitions, signal transduction, and intracellular downstream pathways (14, 22). Among these PRRs, CLRs have been shown to be essential for fungal recognition, either alone or in conjunction with TLRs (23–25). The family of CLRs comprises a subset of CTLD-containing proteins, including some Ca^2+^-dependent and Ca^2+^-independent carbohydrate-binding membrane-bound receptors. They are preferentially expressed by myeloid cells (26, 27). Ca^2+^-dependent carbohydrate binding is the most common CTLD function in vertebrates. Under these circumstances, the CTLD is therefore named as a carbohydrate recognition domain (CRD) (27). CLRs can recognize an array of molecules such as carbohydrates, proteins, and lipids.

Several PRRs have been reported to recognize β-glucans, including Dectin-1, complement receptor 3 (CR3), and three members of the scavenger receptor family, CD5, CD36, and SCARF1 (28–33). Recent studies have revealed that Dectin-2, Dectin-3, macrophage mannose receptor (MR), macrophage-inducible C-type lectin (Mincle), and dendritic cell (DC)-specific ICAM3-grabbing non-integrin (DC-SIGN) can recognize mannans and mannoproteins (12, 13, 34–37). It has been shown that Dectin-1 specifically recognizes β-1, 3-glucans (11, 38), whereas Dectin-2 and Dectin-3 specifically recognize α-mannans (12, 13). The receptor(s) that recognizes chitin is still unknown, although NOD2, MR, and TLR9 were proposed to recognize chitin (39).

## CLRs in Antifungal Immune Response

**Figure 1:**
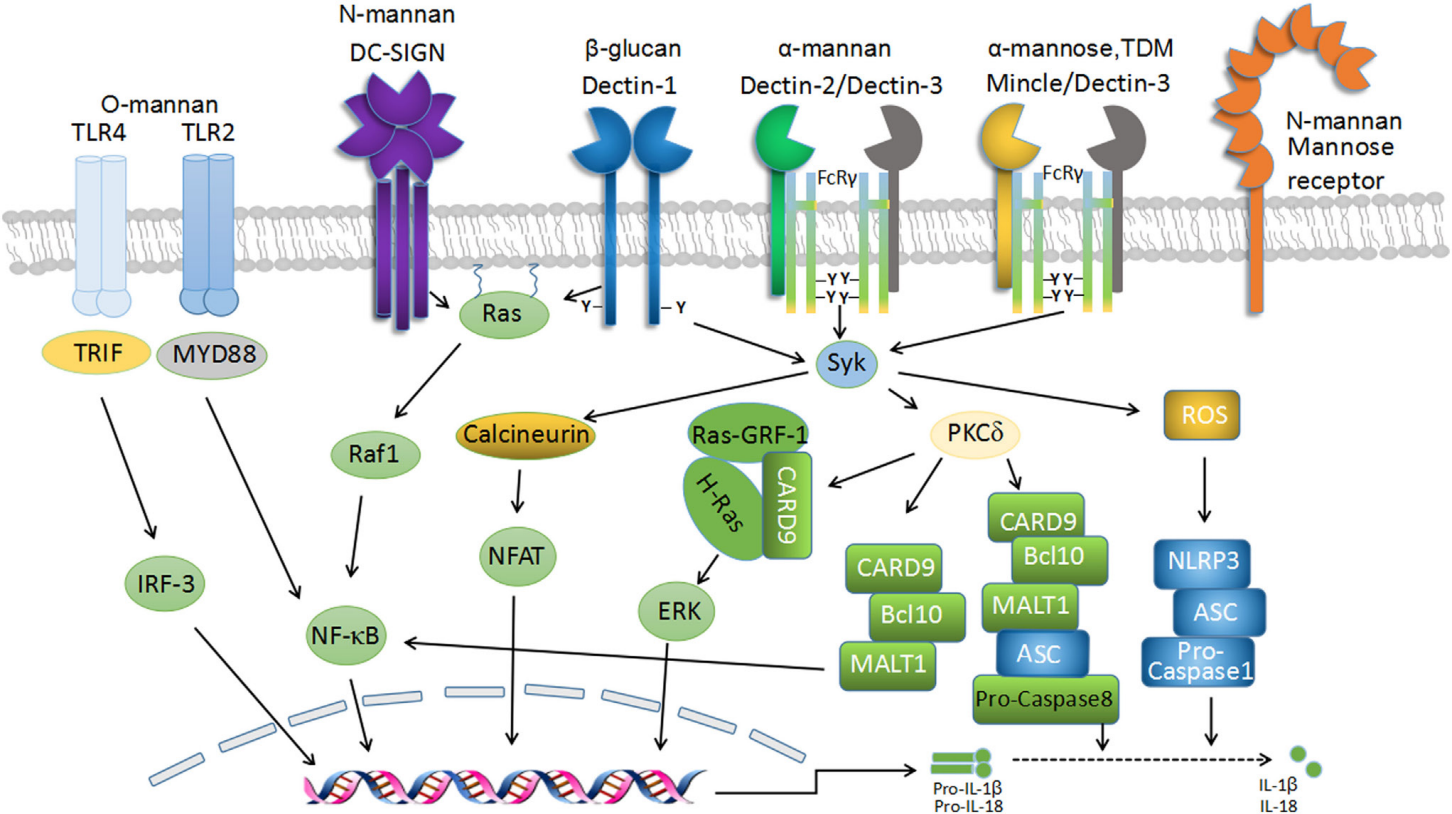
C-type lectin receptor (CLR) signaling in fungal recognition. Schematic representation of the transmembrane CLRs associated with antifungal immunity and their intracellular-signaling networks. The recognition of fungal components by several CLRs, including Dectin-1, Dectin-2, Dectin-3, macrophage-inducible C-type lectin (Mincle), and dendritic cell-specific ICAM3-grabbing non-integrin (DC-SIGN), induces downstream signaling *via* Syk-dependent and Raf-1-dependent pathways to mediate antifungal immunity. Dectin-1 homodimers, Dectin-2-Dectin-3 heterodimers, and Mincle-Dectin-3 heterodimers couple with immunoreceptor tyrosine-based activation motif (ITAM)-like and FcR-γ-associated ITAM motifs, respectively, to recruit Syk to initiate downstream signaling, which leads to reactive oxygen species (ROS) production and caspase activation and recruitment domain-containing protein 9 (CARD9)/Bcl-10/MALT1 complex-mediated activation of NF-κB pathway. ROS triggers NLRP3 inflammasome assembly and activation, which cleave pro-IL-1β and pro-IL-18 into mature forms to elicit protective roles in anti-fungi immunity. Dectin-1-signaling pathway also induces Syk-dependent activation of Ras-GRF1, which recruits H-Ras *via* the CARD9 adaptor and ultimately leads to extracellular signal-regulated protein kinase (ERK) activation. Furthermore, signaling by Syk results in nuclear factor of activated T cells (NF-AT) activation in a calcineurin-dependent fashion, which integrates with NF-κB signaling to regulate gene transcription. Moreover, Dectin-1-signaling activation seems to be essential for the formation of noncanonical caspase-8 inflammasome, which is responsible for active IL-1β production.

The major CLRs that are recognized to be involved in antifungal immune responses are Dectin-1, Dectin-2, Dectin-3, MR, Mincle, and DC-SIGN. Dectin-1, Dectin-2, Dectin-3, Mincle, and DC-SIGN share a similar molecular structure consisting of a CRD, a stalk region, a transmembrane domain, and a cytoplasmic domain (40–42). By contrast, MR is composed of an amino terminal cysteine-rich domain, a single fibronectin type II domain, eight CRDs, a transmembrane domain, and a cytoplasmic tail (43, 44) (Figure 1). These CLRs can be divided into two main groups based on their intracellular-signaling motifs: CLRs with immunoreceptor tyrosine-based activation motifs (ITAMs) or ITAM-like (also named hem-ITAM) domains and CLRs containing non-immunoreceptor tyrosine-based motifs such as MR and DC-SIGN (27, 45). The activation of these receptors can transduce intracellular-signaling pathways directly through integral ITAM-like motif(s) within the cytoplasmic tails (such as Dectin-1), or indirectly through association with ITAM-containing FcR-γ chains, including Dectin-2, Dectin-3, and Mincle (25, 27, 46). Upon ligand binding, the activation of receptors induces the tyrosine phosphorylation of ITAM-like/ITAM motif(s) by Src family kinases, leading to the recruitment and activation of Syk kinase. This subsequently initiates downstream-signaling pathways. The activation of Syk signaling requires its interaction with two phosphorylated tyrosines within ITAM-like/ITAM motifs. However, unlike canonical ITAM motifs within FcR-γ adaptors which contain a repeat of YxxI/L, the ITAM-like motif located in the cytoplasmic tail of Dectin-1 has only a single YxxI/L; thus, the signaling from Syk may involve the receptor dimerization of Dectin-1 containing a single phosphotyrosine (25, 47) (Figure 1). Signals from the CLRs initiate and modulate not only innate immune responses but also the development of adaptive immunity, especially T_H_1 and T_H_17 responses, which are crucial for the control of fungal infections. The role of Dectin-1, Dectin-2, and Dectin-3 in antifungal immunity is of considerable interest and has been extensively studied during the last decade.

### Signaling Pathways Mediated by Dectin-1

Dectin-1 (encoded by *Clec7a*), which is mostly expressed by myeloid phagocytes (macrophages, DCs, and neutrophils), recognizes β-1,3-glucans in a calcium-independent manner (40). The engagement of Dectin-1 by β-1,3-glucans induces the activation of Src protein tyrosine kinase (PTK). Src phosphorylates the single cytoplasmic ITAM-like domain of Dectin-1, which subsequently results in the recruitment and activation of Syk (25, 48, 49). The activated Syk then phosphorylates protein kinase C δ (PKC-δ), which phosphorylates caspase activation and recruitment domain-containing protein 9 (CARD9) (50, 51). This facilitates complex formation with Bcl-10 and MALT1 (51, 52), thus eliciting NF-κB activation (24, 51) (Figure 1). In addition, Dectin-1-induced activation of extracellular signal-regulated protein kinase (ERK) is also mediated through CARD9, which links Ras-GRF1 to H-Ras (53). In addition to the Syk-dependent pathways, signaling from Dectin-1 also involves Syk-independent pathways mediated by Raf-1, resulting in noncanonical NF-κB activation by collaboration with the Syk-dependent NF-κB-inducing kinase (NIK) pathway (24). The activation of NF-κB and ERK mediates the inflammatory responses against fungal infections and directs T_H_1/T_H_17 differentiation for antifungal immunity (24, 54–56).

Dectin-1 signaling induces numerous signaling events characterized by phagocytosis, respiratory burst, and the production of various inflammatory mediators, including cytokines, chemokines, and inflammatory lipids (40, 47, 57, 58). It is well established that the production of IL-1β, together with IL-6 and IL-23, is essential for antifungal immune responses partly through priming adaptive immunity to differentiate CD4^+^ T cells to T_H_1/T_H_17 cells (24, 56, 59). Moreover, the production of type I interferons (IFNs) can be induced after fungal recognition and require Dectin-1/Syk signaling and transcription factor IRF5 involvement (49). In mouse models, enhanced IFN-β secretion is critical for protection from fungal challenge (49, 60). Importantly, consistent with animal studies, type I IFNs exert a protective role against *C. albicans* infection in humans (61). Dectin-1 signaling also triggers the activation of nuclear factor of activated T cells (NF-AT) through the Syk/calcineurin pathway, leading to the production of inflammatory cytokines, such as IL-2, IL-10, and IL-12 p70, and the regulation of T cell development and differentiation (62–64) (Figure 1).

CARD9 is considered to be essential for tailoring immune responses to fungal pathogens (51). The survival of *Card9^–/–^* mice is greatly impaired following systemic *C. albicans* infection (51, 53). In addition, NF-κB-mediated cytokine production is severely defective in the absence of CARD9 (51, 53). Notably, human CARD9 deficiency, which is referred to as an autosomal-recessive disorder, is associated with a spectrum of fungal diseases caused by various fungal pathogens (65). Currently, 16 human CARD9 mutations, including nonsense and missense mutations, have been reported in patients worldwide (66). CARD9 mutations result in the impairment of mucosal fungal defense, partly by inhibiting T_H_17-induced immune responses, which are responsible for the susceptibility to chronic mucocutaneous candidiasis (67). However, the underlying mechanism regarding how human CARD9 mutations affect T_H_17 immunity deserves further investigation.

Strikingly, CARD9 serves as the only currently known human gene in regulating the dissemination of *C. albicans* to the central nervous system (CNS). CARD9 deficiency in both mice and humans results in vulnerability to fungal infection in the CNS, owing to impaired neutrophil accumulation in the fungal-infected CNS, which correlates with the lack of CXC-chemokine induction (68). Consistent with these findings, decreased neutrophil recruitment to the lungs was reported in *Card9^–/–^* mice infected with *A. fumigatus* (69). CARD9 deficiency may also predispose to extrapulmonary *A. fumigatus* infection in humans as a result of impaired neutrophil recruitment (70). Nevertheless, the CARD9-dependent-protective role seems less necessary for pulmonary mold infections. Thus, future studies are required to decipher the role of CARD9 in other immune cells to explain its “organ-specific” and “species-specific” function in antifungal immunity. Moreover, the effects of genetic mutations in CARD9-coupled receptors (Dectin-1, Dectin-2, and Dectin-3) and CARD9-binding partners (MALT1 and Bcl-10) in human antifungal host defense require more in-depth studies.

Vav proteins, the key upstream regulators of CARD9, are critical in CLR/CARD9-induced-inflammatory responses similar to CARD9 (71). Indeed, humans with polymorphisms in *DECTIN-1 and VAV3* show increased susceptibility to invasive *C. albicans* infection (71, 72). Interestingly, one recent study illustrated that neutrophilic myeloid-derived suppressor cells (MDSCs) are induced *in vitro* upon infection with various *Candida* species, which functionally inhibit T cell responses *via* Dectin-1/CARD9 signaling and subsequently suppress ROS generation, indicating that CARD9 seems to function as a negative modulator in fungal immune response (73). It is unknown whether this is true *in vivo*. The contribution of MDSCs to fungal infections requires further investigation.

### Signaling Pathways Mediated by Dectin-2, Dectin-3, and Mincle

Dectin-2 (encoded by *Clec4n*), Dectin-3 (MCL, encoded by *Clec4d*), and Mincle (encoded by *Clec4e*) belong to the Dectin-2 family of CLRs, whose encoding genes are grouped closely at the telomeric end of the NK-gene cluster. They all have a single extracellular CTLD, short cytoplasmic tails, and trigger intracellular signaling indirectly through association with the ITAM-containing FcR-γ chain (55, 74–76). Signaling from Dectin-2, Dectin-3, and Mincle is mediated *via* the Syk/PKCδ-dependent CARD9/Bcl-10/MALT1 pathway, resulting in the activation of the transcription factor NF-κB and the subsequent production of inflammatory cytokines and chemokines (13, 75, 77). Dectin-2 recognizes high-mannose structures and binds *Candida* α-mannans in a calcium-dependent manner (12, 78). It can also recognize O-linked mannobiose-rich glycoprotein from *Malassezia*, glycans containing mannose from house dust mite extracts (19, 79). Dectin-2 has been implicated in the defense against numerous pathogens, including *C. albicans, C. neoformans, A. fumigatus, Saccharomyces cerevisiae, Paracoccidioides brasiliensis, Histoplasma capsulatum, Microsporum audouinii, Trichophyton rubrum, Mycobacterium tuberculosis*, and *Schistosoma mansoni* (75, 78, 80). Dectin-2 and Dectin-3 can form heterodimers to recognize the hyphal forms of *C. albicans* to induce pro-inflammatory production (13), although the involvement of Dectin-3 in *C. neoformans* infection is still controversial (81).

Mice deficient for Dectin-2 are highly susceptible to systemic candidiasis (12). Further study indicates that Dectin-2 and Dectin-3, two similar CLRs, form a constitutive heterodimeric PRR for sensing α-mannans on the surface of *C. albicans* and induce Syk-mediated activation of NF-κB to combat fungal invasion (13). Blocking either Dectin-2 or Dectin-3 with antibodies dramatically eliminates NF-κB-mediated-inflammatory responses upon *C. albicans* stimulation. The genetic deletion of Dectin-3, or mice receiving Dectin-3-blocking antibodies, showed high susceptibility to systemic candidiasis (13). Therefore, Dectin-2 coupled with Dectin-3 displays protective antifungal immunity in animal models. Recently, two studies also showed that Dectin-3 is constitutively expressed in myeloid cells and functions as an FcR-γ-coupled receptor for sensing trehalose-6,6’-dimycolate (TDM), a potent mycobacterial adjuvant (76, 82). In addition, Dectin-3 is also essential for inducing Mincle expression upon TDM stimulation (82). Dectin-3 has been shown to interact with Mincle *via* the stalk region of Dectin-3, thus enhancing the protein expression of Mincle (83).

Emerging evidence showed that the engagement of the T_H_17/IL-17 pathway plays a critical role in host defense against mucosal fungal infection (84). Both Dectin-2 and Dectin-3 are of great importance for T_H_17 cell differentiation in host defense against *C. albicans* or *Blastomyces dermatitidis* (12, 85). Furthermore, PI3K-δ, a proximal Syk-dependent-signaling intermediate downstream of Dectin-2, plays an important role in the generation of T_H_2 and T_H_17 immunities against infection with *Dermatophagoides farina* (*D. farina*) (86). In addition, a recent study showed that NF-κB subunit c-Rel-dependent cytokine induction relies on the Dectin-2/MALT1-signaling cascade to trigger T_H_17-polarizing cytokines IL-1β and IL-23 secretion, thus possessing T_H_17-protective immunity against pathogenic fungal invasion (52). The expression of IL-17RC on humans and murine neutrophils has been identified in a Dectin-2-dependent pathway (87). Dectin-2-induced autocrine IL-17 secretion has also been implicated with ROS generation and fungal killing (87).

Mincle has been shown to recognize *mycobacteria, C. albicans, Malasezzia*, and *Fonsecaea species* (36, 88–90). It is the sensor for α-mannose, glycolipid trehalose-6, 6′-dimycolate (TDM), and the self-ribonucleoprotein SAP-130 (74, 88, 91). Mincle is expressed constitutively at low levels in myeloid cells, and its expression is dependent on Dectin-3 (76, 83, 92). The expression pattern of Mincle suggests that it may not be the major fungal recognition receptor. In support of this notion, although mice lacking Mincle display increased fungal burden in the kidneys, the survival rate of *Mincle*^–/–^ mice is similar to wild-type mice upon systemic *C. albicans* infection (89). It has been shown that Mincle is not a phagocytic receptor but modestly potentiates pro-inflammatory cytokine production (89). Mincle has been shown to inhibit Dectin-1-induced T_H_1 responses to *F. monophora* infection by inducing IRF1 degradation through the E3 ubiquitin ligase Mdm2, which impairs the polarization of T_H_1 cells. Defective T_H_1 responses contribute to the chronic infection of *F. monophora* which causes chromoblastomycosis, a chronic fungal skin infection (93). In addition, Mincle has been demonstrated to specifically recognize *Malassezia* species and play a crucial role in host defense against this fungus (36).

### Other Fungal Recognition Receptors

MR (CD206, encoded by *Mrc1*): MR recognizes N-linked mannan of infectious *Candida* and mediates endocytosis and phagocytosis (94). Recent studies indicate that MR might promote the secretion of pro-inflammatory cytokines through the activating intracellular signal cascades. Although non-ITAM motifs are identified within the MR, a recent study reports that human MR becomes tyrosine phosphorylated upon *M. tuberculosis* (*M. tb*) infection, and this phosphorylation mediates a sequential association of Grb-2 and SHP-1 (95), suggesting that human MR itself can transduce downstream signaling. However, no known signaling motifs in murine MR have been identified, and no signaling has been induced directly from murine MR in response to fungal infections. Using human peripheral blood mononuclear cells, MR was found to be the main receptor pathway for the induction of T_H_17 cells by *C. albicans in vitro* (34). However, the importance of MR in fungal recognition is challenged by the fact that normal host defense is not altered during systemic candidiasis or *Pneumocystis carinii* infection in *Mr*^–/–^ mice (96). In support of this, MR is also not required for resistance to *Coccidioides immitis* infection (97). Therefore, MR may not be the major fungal recognition receptor in mice. It is possible that the human and murine MRs may behave differently. This notion is supported by a recent report that human but not mouse MR signaling induced by *M. tb* regulates macrophage recognition and vesicle trafficking (95).

DC-SIGN (encoded by *Cd209a*): DC-SIGN is a transmembrane receptor for pathogen binding and uptake, which is mainly expressed in a subset of macrophages and DCs (98, 99). It has been demonstrated that DC-SIGN can bind and internalize soluble ligands effectively, which facilitates antigen processing and presentation to T cells (100). DC-SIGN has a high affinity to detect varied carbohydrate-based ligands, including mannose structures and fucose-bearing glycans, to recognize diverse organisms including HIV-1, *M. tb, Helicobacter pylori*, or fungi such as *C. albicans, A. fumigatus*, and *C. tropicum* (35, 37, 101–103). The polymorphisms of both Dectin-1 and DC-SIGN were reported to associate with invasive pulmonary Aspergillosis infection (104). It has been demonstrated that DC-SIGN can recognize *Candida* mannan and that *N*-linked mannosyl residues are essential for this interaction (37). In particular, the *N*-mannosylation is required for the binding, phagocytosis, and immune sensing of *C. albicans* by human DCs (37). Upon high-mannose recognition, the signalosome leads to Raf-1 activation and subsequent p65 acetylation, which facilitates gene-transcriptional expression, especially amplifying TLR-induced cytokine production such as IL-10, IL-6, and IL-12 (23). However, DC-SIGN in collaboration with MR seems to suppress Dectin-1-mediated T_H_17 responses, but potentiate T_H_1 responses in β-glucan- or *M. tb*-treated DCs (105). Moreover, DC-SIGN, which contains abundant galactomannan, is also found to play an important role in the recognition and binding of *A. fumigatus* conidia in human DCs (103). The ligation of DC-SIGN by the glycoprotein fimbriae of *Porphyromonas gingivalis* promotes the evasion of antibacterial autophagy and lysosome fusion, resulting in intracellular persistence in myeloid DCs, whereas TLR2 activation can overcome autophagy evasion and pathogen persistence in DCs (106). However, the importance of DC-SIGN in antifungal immunity has not been verified by a gene-targeting approach.

**Figure 2:**
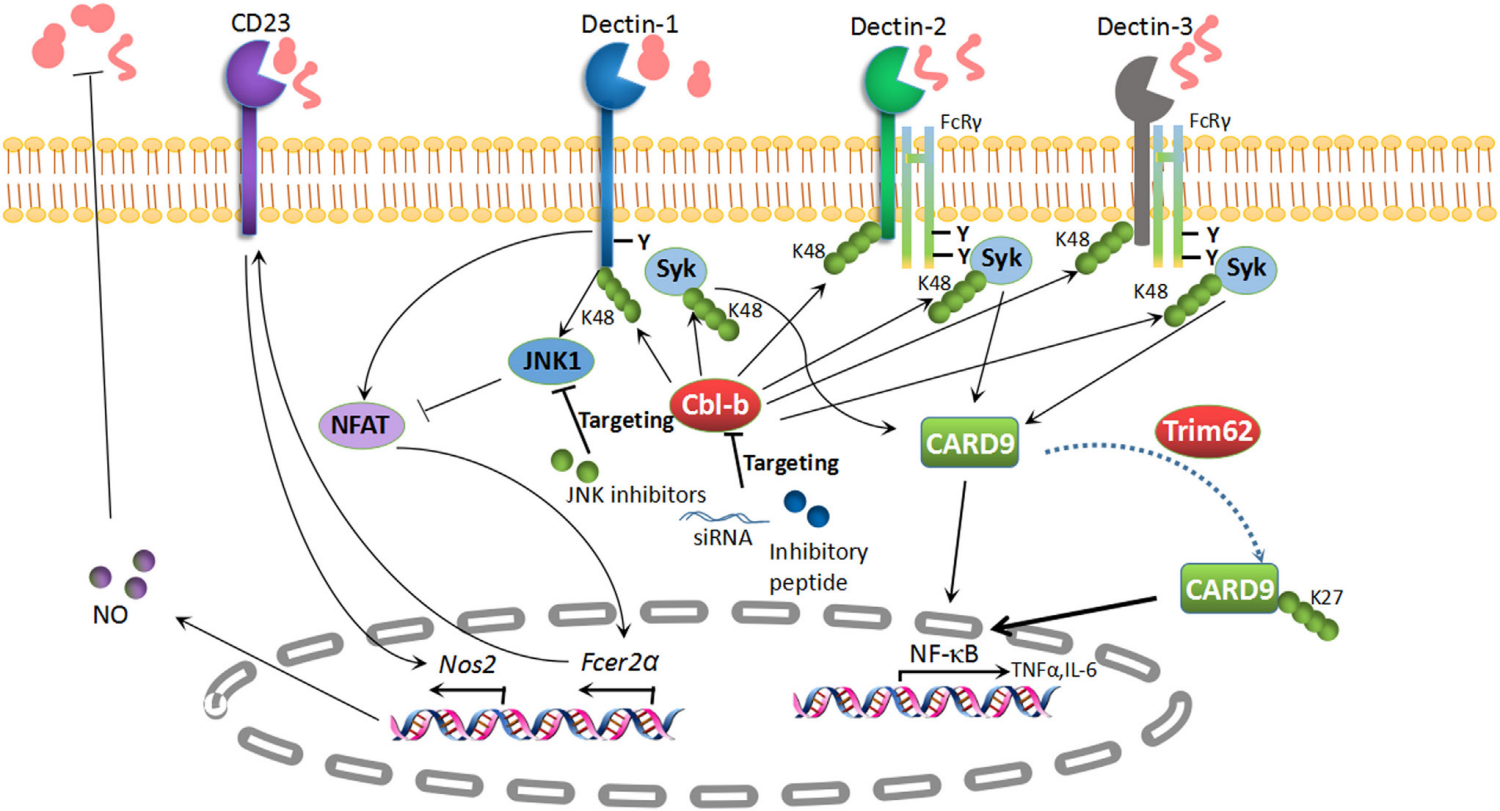
Potential targets in antifungal immunity. K48-linked polyubiquitination of Dectin-1, Dectin-2, Dectin-3, and Syk is mediated by the E3 ubiquitin ligase Cbl-b, which results in the degradation of these molecules. Subsequently, the pro-inflammatory cytokines are impaired in the presence of Cbl-b. The inhibition of Cbl-b with an inhibitory peptide or *Cbl-b* siRNA can boost antifungal immunity, which provides a potential treatment strategy for fungal invasion. CD23 is a novel C-type lectin receptor for fungal recognition, which is elevated in parallel with NO production in *Jnk1^–/–^* mice. The inhibition of c-Jun N-terminal kinase 1 (JNK1) has revealed its potential as a therapeutic target for enhancing antifungal immunity. TRIM62, another E3 ubiquitin ligase, is responsible for K27-linked polyubiquitination of caspase activation and recruitment domain-containing protein 9 (CARD9) to elicit its antifungal immunity. TRIM62 or CARD9 variants appear to be potential therapeutic targets for fungal infections.

CD23 (encoded by *Fcer2a*): CD23 is the low-affinity receptor for IgE and is also a novel CLR which binds to α-mannans and β-glucans (107). A recent study illustrated that c-Jun N-terminal kinase 1 (JNK1) deficiency exerts a protective effect in systemic candidiasis. The expression of CD23 is negatively regulated through a Dectin-1-induced NF-AT pathway (107). Antifungal effector NOS2 is dramatically augmented through the recognition of α-mannans and β-glucans with CD23 in mice lacking JNK1. Likewise, the genetic deletion of CD23 abrogates the protection of *Jnk1^–/–^* mice from disseminated candidiasis. JNK inhibitors boost antifungal innate immunity *in vivo* and *in vitro* (107). Taken together, JNK inhibition may also be a novel therapeutic strategy to combat disseminated candidiasis (Figure 2).

CR3 (Mac-1, α_Mβ2_, or CD11b/CD18): CR3 is mainly expressed in leukocytes. CR3 consists of an I domain and a specific lectin domain, which bind to protein ligands such as iC3b, fibronectin and ICAM-1, and complement deposited on β-1,6-glucans (108). CR3 has been shown to cooperate with Dectin-1 for the detection of β-glucans and the regulation of innate immune responses during fungal pathogen exposure (109). Recent studies identified that CR3 and Dectin-1 collaboratively induce cytokine responses in macrophages in an Syk/JNK/AP-1 manner upon disseminated *H. capsulatum* infection (110), which further facilitates fungal-adaptive immune responses. Still, the underlying molecular mechanisms of crosstalk among other fungal PRRs will be of great interest for future investigations.

### Collaboration between CLRs and TLRs

There is emerging evidence that signaling from CLRs in collaboration with other PRRs, especially TLRs, is indispensable for optimal antifungal immunity. It has been reported that the cooperative interaction between Dectin-1 and TLR2 or TLR4 synergistically facilitates the production of TNF, IL-23, and IL-10, but reduces IL-12 (59, 111). Dectin-1/TLR2 can amplify MR-mediated Th17 responses and IL-17 production upon *C. albicans* infection (34). In addition, DC-SIGN modulates the signaling from multiple TLRs on human DCs through activating Raf-1-dependent acetylation of NF-κB, which can promote the transcription of IL-10 and enhance antifungal-inflammatory response (23).

Recent studies have shown the importance of costimulation of Mincle and TLRs in protective antifungal response to *F. pedrosoi*, the most common fungus associated with chromoblastomycosis. Normally, *F. pedrosoi* is recognized by CLRs, but not TLRs, leading to the defective production of costimulatory cytokines and impaired fungal clearance. Intriguingly, the exogenous application of TLR7 ligand, imiquimod, restores the induction of inflammatory responses mediated *via* both Syk/CARD9- and MyD88-dependent-signaling pathways, as well as facilitates *F. pedrosoi* clearance in mice (90). In support of this finding, the topical administration of imiquimod to several patients with chromoblastomycosis also results in rapid infection resolution and greatly improved the lesions (112).

## Inflammasomes in Antifungal Immunity

Emerging evidence shows that the engagement of inflammasomes plays a critical role in host defense against fungal infection, which can lead to the processing and activation of IL-1β and IL-18 (113). Both cytokines are implicated in mediating antifungal cellular responses, especially the promotion of adaptive T_H_1/T_H_17 responses.

NLRP3 has been proposed to be the main inflammasome involved in protective fungal immunity (114). Several CLRs and TLRs can induce the priming of inflammasomes and the activation of NF-κB *via* the recognition of fungal PAMPs, resulting in the expression of pro-IL-1β and pro-IL-18 (115–117). Both Dectin-1/Syk- and TLR2/MYD88-signaling pathways have been shown to induce NLRP3 priming in murine macrophages infected with *C. albicans* (114). In addition, the production of pro-IL-1β in response to *A. fumigatus, M. canis, Malassezia* spp., *P. brasiliensis*, and *C. neoformans* requires Dectin-1/Syk-dependent signaling (118–122). A more recent study indicates that Dectin-2 is the primary receptor for NLRP3 inflammasome activation in DCs in response to *H. capsulatum* (117). It is unknown whether Dectin-2 and other CLRs such as Dectin-3, Mincle, and MR are also involved in the activation of inflammasomes.

The canonical NLRP3 inflammasome can be triggered by ROS, K^+^ efflux, and lysosomal cathepsins release induced by various fungal species. Upon infection with *C. albicans* and *A. fumigatus*, it has been shown that the activation of the NLRP3 inflammasome requires transition from the yeast to the filamentous phase (123), which may be attributed to the differential exposure of β-glucans on the fungal surface and thus the differential recognition by Dectin-1 (124). Upon phagocytosis by host macrophages, *C. albicans* filaments trigger lysosomal rupture, which is required for the particulate activation of the NLRP3 inflammasome (124, 125). In addition, *C. albicans-*secreted aspartic proteases Sap2 and Sap6 are thought to activate the caspase-1-dependent NLRP3 inflammasome by inducing ROS production and K^+^ efflux (126). Recent evidence has shown that NLRP3 coupling with AIM2 receptors is required to activate caspase-1- and caspase-8-dependent inflammasomes and induce protective antifungal responses in DCs challenged with *A. fumigatus* (127). Mice deficient in both NLRP3 and AIM2 are more susceptible to invasive Aspergillosis than mice lacking a single inflammasome receptor, suggesting the importance of cooperative activation and dual cytoplasmic surveillance of these two inflammasomes against *A. fumigatus* infection (127). Interestingly, mucosal *Candida* infection induces the activation of an NLRC4-dependent inflammasome, which can utilize caspase-1 to process IL-1β and IL-18 (128). The NLRC4 inflammasome protects against mucosal fungal overgrowth and facilitates inflammatory cytokine secretion and neutrophil influx in a murine model of oropharyngeal candidiasis (128).

Recently, an NLR-independent and caspase-8-dependent inflammasome have been identified (115). It seems that Dectin-1 signaling induces the formation of a CARD9/Bcl-10/MALT1/caspase-8/ASC complex which is dependent on Syk (115). Interestingly, caspase-8 in this complex is only partially cleaved to generate a p43 intermediate, which averts the triggering of caspase-3 and apoptosis (115). Dectin-1-mediated activation of caspase-8 appears to be involved in the cleavage of pro-IL-1β and the production of its bioactive form to defend against fungi (115). A subsequent study reported that this noncanonical caspase-8 inflammasome can be activated and modulated by Tec, an intracellular non-receptor PTK, which acts as a novel signaling mediator between Dectin-1/Syk and PLC-γ2 in macrophages upon infection with *Candida* (116). The genetic ablation or the chemical inhibition of Tec results in a dramatic reduction of inflammatory responses and protects from fatal fungal sepsis (116). Interestingly, it has been shown that caspase-8, coordinating with caspase-1, plays a crucial role in promoting NLRP3 inflammasome-dependent maturation of IL-1β mediated by Dectin-1 and CR3 in DCs during β-glucan sensing and *C. albicans* infection (109). In addition, the same group also showed that there is crosstalk between CR3 and Dectin-1 during *H. capsulatum* yeast infection in macrophage TNF and IL-6 responses in an Syk/JNK/AP-1-dependent manner (110). However, it was reported that *H. capsulatum* α-(1,3)-glucan blocks innate immune recognition by Dectin-1 (129). The importance of Dectin-1 in *H. capsulatum* infection, in particular *in vivo*, remains to be determined. Furthermore, the role of caspase-8 in controlling antifungal immunity has not been confirmed by a gene-targeting approach.

## CLR-Mediated PTMS in Antifungal Immunity

Recent literature has shed additional light on novel molecules engaged in antifungal immunity and PTMs in CLR-signaling cascades, thus opening new avenues for innovative therapeutic approaches (107, 130–133). It has been increasingly recognized that PTMs serve as modulators to tailor fungal evasion by targeting innate sensors, adaptors, signaling components, and transcription factors. Subsequently, PTMs regulate the activation, survival, and stability of potent proteins by linking covalent bonds to functional groups (134). To date, several PTMs including phosphorylation and ubiquitination have been characterized in the regulation of immune responses against fungi.

### Protein Kinases and Phosphatases in Antifungal Innate Immunity

Two major cytoplasmic kinase families in innate cells, including the Src family kinases and the Syk, are involved in intracellular-signaling cascades upon fungal pathogen exposure. Signaling involving the phosphorylation of tyrosine residues within the ITAM(s) by Src family kinases leads to the recruitment and activation of Syk, which then phosphorylates phospholipase Cγ2 (PLC-γ2). Activated PLC-γ2 initiates the hydrolysis of membrane-bound phosphatidylinositol-3,4,5-triphosphate (PIP_3_) to soluble inositol triphosphate (IP_3_) and diacylglycerol, both of which result in the influx of calcium and the activation of PKC-δ, the latter mediating the phosphorylation of CARD9 and the subsequent activation of the CARD9/Bcl-10/MALT1 complex. Downstream signaling through the Syk/PLCγ2 pathway from Dectin-1 and Dectin-2 involves the activation of NF-κB, ERK, and NF-AT (24, 53, 135, 136).

Numerous studies have shown that the balance between phosphorylation and dephosphorylation is of great importance in orchestrating fungal immune responses. In addition to Src kinases phosphorylating ITAM(s) within Dectin-1 and FcR-γ, recent literature has shown that two members of Src family kinases, Fyn and Lyn, facilitate the *cryptococcal*-killing capacity in NK cells by mediating PI3K/ERK1/2-signaling activity, which further directs the traffic of perforin-containing granules to synapses for pathogen clearance (137). Whether other Src family kinases are involved in the regulation of fungal invasion remains to be determined.

The CARD9/Bcl-10/MALT1 complex in Dectin-1 signaling upon *Candida* infection has been known to activate the IKK complex, leading to the phosphorylation of IκB and the activation of all canonical NF-κB subunits including p50, p65, and c-Rel (24, 51, 138). Importantly, Syk activation in response to Dectin-1 stimulation is also able to activate noncanonical subunits of NF-κB (p52 and RelB) through NIK and IKKα, leading to the nuclear translocation of p52–RelB dimers (24). In addition, Dectin-1 signaling can induce Syk-independent phosphorylation and activation of Raf-1 *via* Ras. Activated Ras leads to the activation of Raf-1, which then phosphorylates NF-κB p65, and facilitates p65–RelB dimer formation that sequesters active RelB and potentiates T_H_1 responses by inducing IL-12p40 and IL-1β (24). Interestingly, another group recently found that Dectin-1 stimulated with *C. albicans* triggers Syk-dependent phosphorylation of Ras-GRF1, which mediates the recruitment and activation of H-Ras through CARD9 alone, but not the CARD9/Bcl-10/MALT1 complex, leading to the phosphorylation and activation of ERK, but not NF-κB and subsequent pro-inflammatory responses (53). This suggests that upon Dectin-1 signaling, CARD9 is required for ERK activation but is dispensable for NF-κB activation.

CLR (Dectin-1, Dectin-2/3, or Mincle) signaling has been reported to phosphorylate and activate SHP-2, which is able to recruit Syk to Dectin-1 or to the adaptor FcR-γ, thus resulting in the activation of Syk and downstream signaling and mediate antifungal innate immune responses and T_H_17 responses (139). In addition, the phosphatase SHIP-1 has been recently identified to co-localize with Dectin-1-phosphorylated hem-ITAM and to negatively modulate ROS production in a Dectin/Syk/PI3K/PDK1/NADPH oxidase-dependent manner in response to *C. albicans* infection (140). Thus, a novel role of SHIP-1 in selectively controlling the balance of effectors in the Syk/PI3K pathway has been identified. Phosphatase and tensin homolog deleted on chromosome 10 also serves as a negative modulator to regulate the PGE2/cAMP/PKA-signaling cascade *via* blocking F-actin-mediated cytoskeletal remodeling and dephosphorylating cofilin-1 during immune defense against pathogenic *C. albicans* (141). Therefore, phosphorylation- and dephosphorylation-mediated protein kinases and phosphatases are crucial for controlling antifungal immunity.

### Ubiquitin Ligases and Deubiquitinating Enzymes in Antifungal Innate Immunity

PTM of target proteins by polyubiquitination has been intensively studied in numerous biological systems (142–146). Seven lysine residues in ubiquitin determine the specific type of polyubiquitination. Lysine 48 (K48)-linked polyubiquitination is involved in proteasome-mediated protein degradation, whereas lysine 63-linked polyubiquitination is usually engaged in signal pathway transmission (145). In addition to K48- and K63-linked polyubiquitination, K6-, K11-, K27-, K29-, and K33-linked polyubiquitination are being exploited to address their roles in immune responses and inflammatory diseases (145). Recently, a number of studies have highlighted an important role for ubiquitination of the CLR-signaling pathway in fungal immunity (130–133).

#### TRIM62

TRIM62, also named DEAR1, is a member of the TRIM/RBCC family, which includes proteins with conserved RING finger, B-box, and coiled-coil domains (147). It has been well established that CARD9 positively modulates host immune responses following fungal infection. TRIM62 has been shown to function as a CARD9-binding component and to mediate K27-linked polyubiquitination of CARD9 at K125 to facilitate its protective role in anti-fungi immune responses (130). Similar to *Card9^–/–^* mice, *Trim62^–/–^* mice also show increased susceptibility, as well as impaired cytokine responses, in a *C. albicans* infection model (130). Therefore, TRIM62 acts as a positive regulator essential for CARD9-mediated antifungal immunity. TRIM62 or CARD9 variants are therefore potential therapeutic targets for fungal infections (Figure 2).

#### Cbl-b

Cbl-b is a member of Cbl family RING finger E3 ubiquitin ligases (142, 148). Several other groups including ours identified RING finger-type E3 ubiquitin ligase Cbl-b as a key E3 ubiquitin ligase mediating host antifungal innate immunity (131–133). The genetic deletion of Cbl-b renders mice less susceptible to systemic *C. albicans* infection, which is in line with the hyper-production of pro-inflammatory cytokines TNF-α and IL-6, the robust release of reactive oxygen species (ROS), and improved fungal killing. At the molecular level, Cbl-b targets Dectin-1, Dectin-2, Dectin-3, and SYK for K48-linked polyubiquitination and proteasome-mediated degradation, which further facilitates its anti-inflammatory response (131–133). Interestingly, Cbl-b small-inhibitory peptides and *Cbl-b*-specific siRNA provide protective efficacy against disseminated candidiasis (131, 132). Therefore, targeting Cbl-b may be a potential therapeutic strategy for disseminated candidiasis (Figure 2).

#### A20

A20 is a deubiquitination enzyme which is pivotal for tailoring innate immune responses by inhibiting the NF-κB-signaling cascade (149). IKKγ and TRAF6 activities are dampened in a noncatalytic manner by A20 (149, 150). A recent study showed that A20 is removed by autophagy, which further boosts NF-κB capacity in F4/80^hi^ tissue-resident macrophages to facilitate the pathogen clearance during disseminated *Candida* infection (151).

## Conclusion

Much progress has been made to unveil the underlying mechanisms of fungal immunity. CLRs are considered to be pivotal in orchestrating innate and adaptive immunity against fungal pathogens based on animal and some human genetic studies. The discovery of novel molecules such as Cbl-b and JNK in anti-fungi immune responses has laid the foundation for potential treatment strategies. Yet, the exact crosstalk between innate and adaptive antifungal immunities, and the yet-to-be-defined PTMs, needs to be resolved in future studies. Moreover, translational studies of newly identified molecular targets are essential for future clinical application. Thus, the studies described in this review provide direction for the rational design of therapeutic strategies in disseminated candidiasis; however, further translational studies with animal models remain to be performed before moving forward into clinical application.

## Author Contributions

JT, GL, and JZ conceptualized the scope of the review; JT, GL, and JZ wrote the review; and LT and WYL edited the review.

## Conflict of Interest Statement

The authors declare that the research was conducted in the absence of any commercial or financial relationships that could be construed as a potential conflict of interest.

## References

[1] Brown GD Denning DW Levitz SM Tackling human fungal infections. Science (2012) 336(6082):647 10.1126/science.1222236 22582229

[2] Gudlaugsson O Gillespie S Lee K Vande Berg J Hu J Messer S Attributable mortality of nosocomial candidemia, revisited. Clin Infect Dis (2003) 37(9):1172–7. 10.1086/378745 14557960

[3] Alter SJ McDonald MB Schloemer J Simon R Trevino J Common child and adolescent cutaneous infestations and fungal infections. Curr Probl Pediatr Adolesc Health Care (2018) 48(1):3–25. 10.1016/j.cppeds.2017.11.001 29198783

[4] Falagas ME Betsi GI Athanasiou S. Probiotics for prevention of recurrent vulvovaginal candidiasis: a review. J Antimicrob Chemother (2006) 58(2):266–72. 10.1093/jac/dkl246 16790461

[5] Ansari Z Miller D Galor A. Current thoughts in fungal keratitis: diagnosis and treatment. Curr Fungal Infect Rep (2013) 7(3):209–18. 10.1007/s12281-013-0150-110.1007/s12281-013-0150-1 24040467 PMC3768010

[6] Seyedmousavi S Netea MG Mouton JW Melchers WJ Verweij PE de Hoog GS. Black yeasts and their filamentous relatives: principles of pathogenesis and host defense. Clin Microbiol Rev (2014) 27(3):527–42. 10.1128/CMR.00093-13 24982320 PMC4135901

[7] Brown GD Denning DW Gow NA Levitz SM Netea MG White TC. Hidden killers: human fungal infections. Sci Transl Med (2012) 4(165):165rv13. 10.1126/scitranslmed.3004404 23253612

[8] Byrnes EJ III Li W Lewit Y Ma H Voelz K Ren P Emergence and pathogenicity of highly virulent *Cryptococcus gattii* genotypes in the northwest United States. PLoS Pathog (2010) 6(4):e1000850. 10.1371/journal.ppat.1000850 20421942 PMC2858702

[9] Sears D Schwartz BS. *Candida auris*: an emerging multidrug-resistant pathogen. Int J Infect Dis (2017) 63:95–8. 10.1016/j.ijid.2017.08.017 28888662

[10] Geijtenbeek TB Gringhuis SI. Signalling through C-type lectin receptors: shaping immune responses. Nat Rev Immunol (2009) 9(7):465–79. 10.1038/nri2569 19521399 PMC7097056

[11] Taylor PR Tsoni SV Willment JA Dennehy KM Rosas M Findon H Dectin-1 is required for beta-glucan recognition and control of fungal infection. Nat Immunol (2007) 8(1):31–8. 10.1038/Ni1408 17159984 PMC1888731

[12] Saijo S Ikeda S Yamabe K Kakuta S Ishigame H Akitsu A Dectin-2 recognition of alpha-mannans and induction of Th17 cell differentiation is essential for host defense against *Candida albicans*. Immunity (2010) 32(5):681–91. 10.1016/j.immuni.2010.05.001 20493731

[13] Zhu LL Zhao XQ Jiang C You Y Chen XP Jiang YY C-type lectin receptors dectin-3 and dectin-2 form a heterodimeric pattern-recognition receptor for host defense against fungal infection. Immunity (2013) 39(2):324–34. 10.1016/j.immuni.2013.05.017 23911656

[14] Dambuza IM Brown GD. C-type lectins in immunity: recent developments. Curr Opin Immunol (2015) 32:21–7. 10.1016/j.coi.2014.12.002 25553393 PMC4589735

[15] Gow NAR Latge J-P Munro CA The fungal cell wall: structure, biosynthesis, and function. Microbiol Spectr (2017) 5(3). 10.1128/microbiolspec.FUNK-0035-2016 PMC11687499 28513415

[16] Latge JP. The cell wall: a carbohydrate armour for the fungal cell. Mol Microbiol (2007) 66(2):279–90. 10.1111/j.1365-2958.2007.05872.x 17854405

[17] Levitz SM Innate recognition of fungal cell walls. PLoS Pathog (2010) 6(4):e1000758 10.1371/journal.ppat.1000758 20421940 PMC2858700

[18] Bowman SM Free SJ. The structure and synthesis of the fungal cell wall. Bioessays (2006) 28(8):799–808. 10.1002/bies.20441 16927300

[19] Ishikawa T Itoh F Yoshida S Saijo S Matsuzawa T Gonoi T Identification of distinct ligands for the C-type lectin receptors Mincle and dectin-2 in the pathogenic fungus *Malassezia*. Cell Host Microbe (2013) 13(4):477–88. 10.1016/j.chom.2013.03.008 23601109

[20] Gomez BL Nosanchuk JD Melanin and fungi. Curr Opin Infect Dis (2003) 16(2):91–6. 10.1097/01.aco.0000065076.06965.04 12734441

[21] Nosanchuk JD Stark RE Casadevall A. Fungal melanin: what do we know about structure? Front Microbiol (2015) 6:1463. 10.3389/fmicb.2015.01463 26733993 PMC4687393

[22] Kawai T Akira S. The roles of TLRs, RLRs and NLRs in pathogen recognition. Int Immunol (2009) 21(4):317–37. 10.1093/intimm/dxp017 19246554 PMC2721684

[23] Gringhuis SI den Dunnen J Litjens M van Het Hof B van Kooyk Y Geijtenbeek TB. C-type lectin DC-SIGN modulates toll-like receptor signaling *via* Raf-1 kinase-dependent acetylation of transcription factor NF-kappaB. Immunity (2007) 26(5):605–16. 10.1016/j.immuni.2007.03.012 17462920

[24] Gringhuis SI den Dunnen J Litjens M van der Vlist M Wevers B Bruijns SC Dectin-1 directs T helper cell differentiation by controlling noncanonical NF-kappaB activation through Raf-1 and Syk. Nat Immunol (2009) 10(2):203–13. 10.1038/ni.1692 19122653

[25] Rogers NC Slack EC Edwards AD Nolte MA Schulz O Schweighoffer E e Sousa: Syk-dependent cytokine induction by dectin-1 reveals a novel pattern recognition pathway for C type lectins. Immunity (2005) 22(4):507–17. 10.1016/j.immuni.2005.03.004 15845454

[26] Zelensky AN Gready JE. The C-type lectin-like domain superfamily. FEBS J (2005) 272(24):6179–217. 10.1111/j.1742-4658.2005.05031.x 16336259

[27] Sancho D Reis C e Sousa: signaling by myeloid C-type lectin receptors in immunity and homeostasis. Annu Rev Immunol (2012) 30:491–529. 10.1146/annurev-immunol-031210-101352 22224766 PMC4480235

[28] Brown GD Gordon S. Immune recognition. A new receptor for beta-glucans. Nature (2001) 413(6851):36–7. 10.1038/35092620 11544516

[29] Ross GD Cain JA Myones BL Newman SL Lachmann PJ. Specificity of membrane complement receptor type three (CR3) for beta-glucans. Complement (1987) 4(2):61–74. 10.1159/000463010 3040332

[30] Xia Y Vetvicka V Yan J Hanikyrova M Mayadas T Ross GD. The beta-glucan-binding lectin site of mouse CR3 (CD11b/CD18) and its function in generating a primed state of the receptor that mediates cytotoxic activation in response to iC3b-opsonized target cells. J Immunol (1999) 162(4):2281–90. 9973505

[31] Vera J Fenutria R Canadas O Figueras M Mota R Sarrias MR The CD5 ectodomain interacts with conserved fungal cell wall components and protects from zymosan-induced septic shock-like syndrome. Proc Natl Acad Sci U S A (2009) 106(5):1506–11. 10.1073/pnas.0805846106 19141631 PMC2635803

[32] Brown GD Taylor PR Reid DM Willment JA Williams DL Martinez-Pomares L Dectin-1 is a major beta-glucan receptor on macrophages. J Exp Med (2002) 196(3):407–12. 10.1084/Jem.20020470 12163569 PMC2193936

[33] Means TK Mylonakis E Tampakakis E Colvin RA Seung E Puckett L Evolutionarily conserved recognition and innate immunity to fungal pathogens by the scavenger receptors SCARF1 and CD36. J Exp Med (2009) 206(3):637–53. 10.1084/jem.20082109 19237602 PMC2699123

[34] van de Veerdonk FL Marijnissen RJ Kullberg BJ Koenen HJ Cheng SC Joosten I The macrophage mannose receptor induces IL-17 in response to *Candida albicans*. Cell Host Microbe (2009) 5(4):329–40. 10.1016/j.chom.2009.02.006 19380112

[35] Cambi A Gijzen K de Vries J Torensma R Joosten B Adema GJ The C-type lectin DC-SIGN (CD209) is an antigen-uptake receptor for *Candida albicans* on dendritic cells. Eur J Immunol (2003) 33(2):532–8. 10.1002/immu.200310029 12645952

[36] Yamasaki S Matsumoto M Takeuchi O Matsuzawa T Ishikawa E Sakuma M C-type lectin Mincle is an activating receptor for pathogenic fungus, *Malassezia*. Proc Natl Acad Sci U S A (2009) 106(6):1897–902. 10.1073/pnas.0805177106 19171887 PMC2644135

[37] Cambi A Netea MG Mora-Montes HM Gow NA Hato SV Lowman DW Dendritic cell interaction with *Candida albicans* critically depends on N-linked mannan. J Biol Chem (2008) 283(29):20590–9. 10.1074/jbc.M709334200 18482990 PMC2459306

[38] Saijo S Fujikado N Furuta T Chung SH Kotaki H Seki K Dectin-1 is required for host defense against *Pneumocystis carinii* but not against *Candida albicans*. Nat Immunol (2007) 8(1):39–46. 10.1038/ni1425 17159982

[39] Wagener J Malireddi RK Lenardon MD Koberle M Vautier S MacCallum DM Fungal chitin dampens inflammation through IL-10 induction mediated by NOD2 and TLR9 activation. PLoS Pathog (2014) 10(4):e1004050. 10.1371/journal.ppat.1004050 24722226 PMC3983064

[40] Brown GD. Dectin-1: a signalling non-TLR pattern-recognition receptor. Nat Rev Immunol (2006) 6(1):33–43. 10.1038/Nri1745 16341139

[41] Graham LM Brown GD. The dectin-2 family of C-type lectins in immunity and homeostasis. Cytokine (2009) 48(1–2):148–55. 10.1016/j.cyto.2009.07.010 19665392 PMC2756403

[42] Guo Y Feinberg H Conroy E Mitchell DA Alvarez R Blixt O Structural basis for distinct ligand-binding and targeting properties of the receptors DC-SIGN and DC-SIGNR. Nat Struct Mol Biol (2004) 11(7):591–8. 10.1038/nsmb784 15195147

[43] Kruskal BA Sastry K Warner AB Mathieu CE Ezekowitz RA. Phagocytic chimeric receptors require both transmembrane and cytoplasmic domains from the mannose receptor. J Exp Med (1992) 176(6):1673–80. 10.1084/jem.176.6.1673 1460425 PMC2119468

[44] Boskovic J Arnold JN Stilion R Gordon S Sim RB Rivera-Calzada A Structural model for the mannose receptor family uncovered by electron microscopy of Endo180 and the mannose receptor. J Biol Chem (2006) 281(13):8780–7. 10.1074/jbc.M513277200 16452473

[45] Svajger U Anderluh M Jeras M Obermajer N. C-type lectin DC-SIGN: an adhesion, signalling and antigen-uptake molecule that guides dendritic cells in immunity. Cell Signal (2010) 22(10):1397–405. 10.1016/j.cellsig.2010.03.018 20363321 PMC7127357

[46] Kerrigan AM Brown GD Syk-coupled C-type lectins in immunity. Trends Immunol (2011) 32(4):151–6. 10.1016/j.it.2011.01.002 21334257 PMC3074083

[47] Goodridge HS Reyes CN Becker CA Katsumoto TR Ma J Wolf AJ Activation of the innate immune receptor dectin-1 upon formation of a ‘phagocytic synapse’. Nature (2011) 472(7344):471–5. 10.1038/nature10071 21525931 PMC3084546

[48] Leibundgut-Landmann S Osorio F Brown GD Reis C e Sousa: stimulation of dendritic cells *via* the dectin-1/Syk pathway allows priming of cytotoxic T-cell responses. Blood (2008) 112(13):4971–80. 10.1182/blood-2008-05-158469 18818389

[49] del Fresno C Soulat D Roth S Blazek K Udalova I Sancho D Interferon-beta production *via* dectin-1-Syk-IRF5 signaling in dendritic cells is crucial for immunity to *C. albicans*. Immunity (2013) 38(6):1176–86. 10.1016/j.immuni.2013.05.010 23770228

[50] Strasser D Neumann K Bergmann H Marakalala MJ Guler R Rojowska A Syk kinase-coupled C-type lectin receptors engage protein kinase C-sigma to elicit Card9 adaptor-mediated innate immunity. Immunity (2012) 36(1):32–42. 10.1016/j.immuni.2011.11.015 22265677 PMC3477316

[51] Gross O Gewies A Finger K Schafer M Sparwasser T Peschel C Card9 controls a non-TLR signalling pathway for innate anti-fungal immunity. Nature (2006) 442(7103):651–6. 10.1038/nature04926 16862125

[52] Gringhuis SI Wevers BA Kaptein TM van Capel TM Theelen B Boekhout T Selective C-Rel activation *via* Malt1 controls anti-fungal T(H)-17 immunity by dectin-1 and dectin-2. PLoS Pathog (2011) 7(1):e1001259. 10.1371/journal.ppat.1001259 21283787 PMC3024268

[53] Jia XM Tang B Zhu LL Liu YH Zhao XQ Gorjestani S CARD9 mediates dectin-1-induced ERK activation by linking Ras-GRF1 to H-Ras for antifungal immunity. J Exp Med (2014) 211(11):2307–21. 10.1084/jem.20132349 25267792 PMC4203953

[54] LeibundGut-Landmann S Gross O Robinson MJ Osorio F Slack EC Tsoni SV e Sousa: Syk- and CARD9-dependent coupling of innate immunity to the induction of T helper cells that produce interleukin 17. Nat Immunol (2007) 8(6):630–8. 10.1038/ni1460 17450144

[55] Robinson MJ Osorio F Rosas M Freitas RP Schweighoffer E Gross O e Sousa: dectin-2 is a Syk-coupled pattern recognition receptor crucial for Th17 responses to fungal infection. J Exp Med (2009) 206(9):2037–51. 10.1084/jem.20082818 19703985 PMC2737172

[56] Zielinski CE Mele F Aschenbrenner D Jarrossay D Ronchi F Gattorno M Pathogen-induced human TH17 cells produce IFN-gamma or IL-10 and are regulated by IL-1beta. Nature (2012) 484(7395):514–8. 10.1038/nature10957 22466287

[57] Goodridge HS Underhill DM Touret N. Mechanisms of Fc receptor and dectin-1 activation for phagocytosis. Traffic (2012) 13(8):1062–71. 10.1111/j.1600-0854.2012.01382.x 22624959

[58] Underhill DM Rossnagle E Lowell CA Simmons RM. Dectin-1 activates Syk tyrosine kinase in a dynamic subset of macrophages for reactive oxygen production. Blood (2005) 106(7):2543–50. 10.1182/blood-2005-03-1239 15956283 PMC1895265

[59] Dennehy KM Willment JA Williams DL Brown GD. Reciprocal regulation of IL-23 and IL-12 following co-activation of dectin-1 and TLR signaling pathways. Eur J Immunol (2009) 39(5):1379–86. 10.1002/Eji.200838543 19291703 PMC2720084

[60] Bourgeois C Majer O Frohner IE Lesiak-Markowicz I Hildering KS Glaser W Conventional dendritic cells mount a type I IFN response against *Candida* spp. requiring novel phagosomal TLR7-mediated IFN-beta signaling. J Immunol (2011) 186(5):3104–12. 10.4049/jimmunol.1002599 21282509

[61] Smeekens SP Ng A Kumar V Johnson MD Plantinga TS van Diemen C Functional genomics identifies type I interferon pathway as central for host defense against *Candida albicans*. Nat Commun (2013) 4:1342. 10.1038/ncomms2343 23299892 PMC3625375

[62] Goodridge HS Simmons RM Underhill DM. Dectin-1 stimulation by *Candida albicans* yeast or zymosan triggers NFAT activation in macrophages and dendritic cells. J Immunol (2007) 178(5):3107–15. 10.4049/jimmunol.178.5.3107 17312158

[63] Fric J Zelante T Wong AY Mertes A Yu HB Ricciardi-Castagnoli P NFAT control of innate immunity. Blood (2012) 120(7):1380–9. 10.1182/blood-2012-02-404475 22611159

[64] Macian F. NFAT proteins: key regulators of T-cell development and function. Nat Rev Immunol (2005) 5(6):472–84. 10.1038/nri1632 15928679

[65] Lanternier F Pathan S Vincent QB Liu L Cypowyj S Prando C Deep dermatophytosis and inherited CARD9 deficiency. N Engl J Med (2013) 369(18):1704–14. 10.1056/NEJMoa1208487 24131138 PMC4084693

[66] Drummond RA Lionakis MS. Mechanistic insights into the role of C-type lectin receptor/CARD9 signaling in human antifungal immunity. Front Cell Infect Microbiol (2016) 6:39. 10.3389/fcimb.2016.00039 27092298 PMC4820464

[67] Glocker EO Hennigs A Nabavi M Schaffer AA Woellner C Salzer U A homozygous CARD9 mutation in a family with susceptibility to fungal infections. N Engl J Med (2009) 361(18):1727–35. 10.1056/NEJMoa0810719 19864672 PMC2793117

[68] Drummond RA Collar AL Swamydas M Rodriguez CA Lim JK Mendez LM CARD9-dependent neutrophil recruitment protects against fungal invasion of the central nervous system. PLoS Pathog (2015) 11(12):e1005293. 10.1371/journal.ppat.1005293 26679537 PMC4683065

[69] Jhingran A Kasahara S Shepardson KM Junecko BA Heung LJ Kumasaka DK Compartment-specific and sequential role of MyD88 and CARD9 in chemokine induction and innate defense during respiratory fungal infection. PLoS Pathog (2015) 11(1):e1004589. 10.1371/journal.ppat.1004589 25621893 PMC4306481

[70] Rieber N Gazendam RP Freeman AF Hsu AP Collar AL Sugui JA Extrapulmonary *Aspergillus* infection in patients with CARD9 deficiency. JCI Insight (2016) 1(17):e89890. 10.1172/jci.insight.89890 27777981 PMC5070961

[71] Roth S Bergmann H Jaeger M Yeroslaviz A Neumann K Koenig PA Vav proteins are key regulators of Card9 signaling for innate antifungal immunity. Cell Rep (2016) 17(10):2572–83. 10.1016/j.celrep.2016.11.018 27926862 PMC5177621

[72] Fischer M Spies-Weisshart B Schrenk K Gruhn B Wittig S Glaser A Polymorphisms of dectin-1 and TLR2 predispose to invasive fungal disease in patients with acute myeloid leukemia. PLoS One (2016) 11(3):e0150632. 10.1371/journal.pone.0150632 26963509 PMC4786091

[73] Rieber N Singh A Oz H Carevic M Bouzani M Amich J Pathogenic fungi regulate immunity by inducing neutrophilic myeloid-derived suppressor cells. Cell Host Microbe (2015) 17(4):507–14. 10.1016/j.chom.2015.02.007 25771792 PMC4400268

[74] Yamasaki S Ishikawa E Sakuma M Hara H Ogata K Saito T. Mincle is an ITAM-coupled activating receptor that senses damaged cells. Nat Immunol (2008) 9(10):1179–88. 10.1038/ni.1651 18776906

[75] Sato K Yang XL Yudate T Chung JS Wu J Luby-Phelps K Dectin-2 is a pattern recognition receptor for fungi that couples with the Fc receptor gamma chain to induce innate immune responses. J Biol Chem (2006) 281(50):38854–66. 10.1074/jbc.M606542200 17050534

[76] Miyake Y Toyonaga K Mori D Kakuta S Hoshino Y Oyamada A C-type lectin MCL is an FcRgamma-coupled receptor that mediates the adjuvanticity of mycobacterial cord factor. Immunity (2013) 38(5):1050–62. 10.1016/j.immuni.2013.03.010 23602766

[77] Kerscher B Willment JA Brown GD. The dectin-2 family of C-type lectin-like receptors: an update. Int Immunol (2013) 25(5):271–7. 10.1093/intimm/dxt006 23606632 PMC3631001

[78] McGreal EP Rosas M Brown GD Zamze S Wong SY Gordon S The carbohydrate-recognition domain of dectin-2 is a C-type lectin with specificity for high mannose. Glycobiology (2006) 16(5):422–30. 10.1093/glycob/cwj077 16423983

[79] Barrett NA Maekawa A Rahman OM Austen KF Kanaoka Y. Dectin-2 recognition of house dust mite triggers cysteinyl leukotriene generation by dendritic cells. J Immunol (2009) 182(2):1119–28. 10.4049/jimmunol.182.2.1119 19124755 PMC3682801

[80] Ritter M Gross O Kays S Ruland J Nimmerjahn F Saijo S da Costa: schistosoma mansoni triggers dectin-2, which activates the Nlrp3 inflammasome and alters adaptive immune responses. Proc Natl Acad Sci U S A (2010) 107(47):20459–64. 10.1073/pnas.1010337107 21059925 PMC2996650

[81] Campuzano A Castro-Lopez N Wozniak KL Leopold Wager CM Wormley FL Jr. Dectin-3 is not required for protection against *Cryptococcus neoformans* infection. PLoS One (2017) 12(1):e0169347. 10.1371/journal.pone.0169347 28107361 PMC5249099

[82] Zhao XQ Zhu LL Chang Q Jiang C You Y Luo T C-type Lectin receptor dectin-3 mediates trehalose 6,6’-dimycolate (TDM)-induced Mincle expression through CARD9/Bcl10/MALT1-dependent nuclear factor (NF)-kappaB activation. J Biol Chem (2014) 289(43):30052–62. 10.1074/jbc.M114.588574 25202022 PMC4208012

[83] Miyake Y Masatsugu OH Yamasaki S. C-type lectin receptor MCL facilitates Mincle expression and signaling through complex formation. J Immunol (2015) 194(11):5366–74. 10.4049/jimmunol.1402429 25888641

[84] Hernandez-Santos N Gaffen SL. Th17 cells in immunity to *Candida albicans*. Cell Host Microbe (2012) 11(5):425–35. 10.1016/j.chom.2012.04.008 22607796 PMC3358697

[85] Wang H Li M Lerksuthirat T Klein B Wuthrich M. The C-type lectin receptor MCL mediates vaccine-induced immunity against infection with *Blastomyces dermatitidis*. Infect Immun (2015) 84(3):635–42. 10.1128/IAI.01263-15 26667836 PMC4771354

[86] Lee MJ Yoshimoto E Saijo S Iwakura Y Lin X Katz HR Phosphoinositide 3-kinase delta regulates dectin-2 signaling and the generation of Th2 and Th17 immunity. J Immunol (2016) 197(1):278–87. 10.4049/jimmunol.1502485 27194783 PMC4912906

[87] Taylor PR Roy S Leal SM Jr Sun Y Howell SJ Cobb BA Activation of neutrophils by autocrine IL-17A-IL-17RC interactions during fungal infection is regulated by IL-6, IL-23, RORgammat and dectin-2. Nat Immunol (2014) 15(2):143–51. 10.1038/ni.2797 24362892 PMC3972892

[88] Ishikawa E Ishikawa T Morita YS Toyonaga K Yamada H Takeuchi O Direct recognition of the mycobacterial glycolipid, trehalose dimycolate, by C-type lectin Mincle. J Exp Med (2009) 206(13):2879–88. 10.1084/jem.20091750 20008526 PMC2806462

[89] Wells CA Salvage-Jones JA Li X Hitchens K Butcher S Murray RZ The macrophage-inducible C-type lectin, Mincle, is an essential component of the innate immune response to *Candida albicans*. J Immunol (2008) 180(11):7404–13. 10.4049/jimmunol.180.11.7404 18490740

[90] Sousa Mda G Reid DM Schweighoffer E Tybulewicz V Ruland J Langhorne J Restoration of pattern recognition receptor costimulation to treat chromoblastomycosis, a chronic fungal infection of the skin. Cell Host Microbe (2011) 9(5):436–43. 10.1016/j.chom.2011.04.005 21575914 PMC3098964

[91] Bugarcic A Hitchens K Beckhouse AG Wells CA Ashman RB Blanchard H. Human and mouse macrophage-inducible C-type lectin (Mincle) bind *Candida albicans*. Glycobiology (2008) 18(9):679–85. 10.1093/glycob/cwn046 18509109

[92] Kerscher B Wilson GJ Reid DM Mori D Taylor JA Besra GS Mycobacterial receptor, Clec4d (CLECSF8, MCL), is coregulated with Mincle and upregulated on mouse myeloid cells following microbial challenge. Eur J Immunol (2016) 46(2):381–9. 10.1002/eji.201545858 26558717 PMC4833188

[93] Wevers BA Kaptein TM Zijlstra-Willems EM Theelen B Boekhout T Geijtenbeek TB Fungal engagement of the C-type lectin Mincle suppresses dectin-1-induced antifungal immunity. Cell Host Microbe (2014) 15(4):494–505. 10.1016/j.chom.2014.03.008 24721577

[94] Porcaro I Vidal M Jouvert S Stahl PD Giaimis J. Mannose receptor contribution to *Candida albicans* phagocytosis by murine E-clone J774 macrophages. J Leukoc Biol (2003) 74(2):206–15. 10.1189/jlb.1202608 12885937

[95] Rajaram MVS Arnett E Azad AK Guirado E Ni B Gerberick AD *M. tuberculosis*-initiated human mannose receptor signaling regulates macrophage recognition and vesicle trafficking by FcRgamma-chain, Grb2, and SHP-1. Cell Rep (2017) 21(1):126–40. 10.1016/j.celrep.2017.09.034 28978467 PMC5960073

[96] Lee SJ Zheng NY Clavijo M Nussenzweig MC. Normal host defense during systemic candidiasis in mannose receptor-deficient mice. Infect Immun (2003) 71(1):437–45. 10.1128/IAI.71.1.437-445.2003 12496194 PMC143203

[97] Viriyakosol S Jimenez Mdel P Saijo S Fierer J. Neither dectin-2 nor the mannose receptor is required for resistance to *Coccidioides immitis* in mice. Infect Immun (2014) 82(3):1147–56. 10.1128/IAI.01355-13 24379281 PMC3957980

[98] van Kooyk Y Geijtenbeek TB. DC-SIGN: escape mechanism for pathogens. Nat Rev Immunol (2003) 3(9):697–709. 10.1038/nri1182 12949494

[99] Tassaneetrithep B Burgess TH Granelli-Piperno A Trumpfheller C Finke J Sun W DC-SIGN (CD209) mediates dengue virus infection of human dendritic cells. J Exp Med (2003) 197(7):823–9. 10.1084/jem.20021840 12682107 PMC2193896

[100] Engering A Geijtenbeek TB van Vliet SJ Wijers M van Liempt E Demaurex N The dendritic cell-specific adhesion receptor DC-SIGN internalizes antigen for presentation to T cells. J Immunol (2002) 168(5):2118–26. 10.4049/jimmunol.168.5.2118 11859097

[101] Hodges A Sharrocks K Edelmann M Baban D Moris A Schwartz O Activation of the lectin DC-SIGN induces an immature dendritic cell phenotype triggering Rho-GTPase activity required for HIV-1 replication. Nat Immunol (2007) 8(6):569–77. 10.1038/ni1470 17496896

[102] Gringhuis SI den Dunnen J Litjens M van der Vlist M Geijtenbeek TB. Carbohydrate-specific signaling through the DC-SIGN signalosome tailors immunity to *Mycobacterium tuberculosis*, HIV-1 and *Helicobacter pylori*. Nat Immunol (2009) 10(10):1081–8. 10.1038/ni.1778 19718030

[103] Serrano-Gomez D Leal JA Corbi AL. DC-SIGN mediates the binding of *Aspergillus fumigatus* and keratinophylic fungi by human dendritic cells. Immunobiology (2005) 210(2–4):175–83. 10.1016/j.imbio.2005.05.011 16164024

[104] Sainz J Lupianez CB Segura-Catena J Vazquez L Rios R Oyonarte S Dectin-1 and DC-SIGN polymorphisms associated with invasive pulmonary Aspergillosis infection. PLoS One (2012) 7(2):e32273. 10.1371/journal.pone.0032273 22384201 PMC3288082

[105] Zenaro E Donini M Dusi S. Induction of Th1/Th17 immune response by *Mycobacterium tuberculosis*: role of dectin-1, Mannose receptor, and DC-SIGN. J Leukoc Biol (2009) 86(6):1393–401. 10.1189/jlb.0409242 19773555

[106] El-Awady AR Miles B Scisci E Kurago ZB Palani CD Arce RM Porphyromonas gingivalis evasion of autophagy and intracellular killing by human myeloid dendritic cells involves DC-SIGN-TLR2 crosstalk. PLoS Pathog (2015) 10(2):e1004647. 10.1371/journal.ppat.1004647 25679217 PMC4352937

[107] Zhao X Guo Y Jiang C Chang Q Zhang S Luo T JNK1 negatively controls antifungal innate immunity by suppressing CD23 expression. Nat Med (2017) 23(3):337–46. 10.1038/nm.4260 28112734 PMC5592785

[108] Brown GD. Innate antifungal immunity: the key role of phagocytes. Annu Rev Immunol (2011) 29:1–21. 10.1146/annurev-immunol-030409-101229 20936972 PMC3434799

[109] Ganesan S Rathinam VAK Bossaller L Army K Kaiser WJ Mocarski ES Caspase-8 modulates dectin-1 and complement receptor 3-driven IL-1beta production in response to beta-glucans and the fungal pathogen, *Candida albicans*. J Immunol (2014) 193(5):2519–30. 10.4049/jimmunol.1400276 25063877 PMC4134963

[110] Huang JH Lin CY Wu SY Chen WY Chu CL Brown GD CR3 and dectin-1 collaborate in macrophage cytokine response through association on lipid rafts and activation of Syk-JNK-AP-1 pathway. PLoS Pathog (2015) 11(7):e1004985. 10.1371/journal.ppat.1004985 26132276 PMC4488469

[111] Brown GD Herre J Williams DL Willment JA Marshall ASJ Gordon S Dectin-1 mediates the biological effects of beta-glucans. J Exp Med (2003) 197(9):1119–24. 10.1084/Jem.20021890 12719478 PMC2193964

[112] de Sousa Mda G Belda W Jr Spina R Lota PR Valente NS Brown GD Topical application of imiquimod as a treatment for chromoblastomycosis. Clin Infect Dis (2014) 58(12):1734–7. 10.1093/cid/ciu168 24633683 PMC4036686

[113] Tavares AH Burgel PH Bocca AL Turning up the heat: inflammasome activation by fungal pathogens. PLoS Pathog (2015) 11(7):e1004948 10.1371/journal.ppat.1004948 26204108 PMC4512686

[114] Hise AG Tomalka J Ganesan S Patel K Hall BA Brown GD An essential role for the NLRP3 inflammasome in host defense against the human fungal pathogen *Candida albicans*. Cell Host Microbe (2009) 5(5):487–97. 10.1016/j.chom.2009.05.002 19454352 PMC2824856

[115] Gringhuis SI Kaptein TM Wevers BA Theelen B van der Vlist M Boekhout T Dectin-1 is an extracellular pathogen sensor for the induction and processing of IL-1beta *via* a noncanonical caspase-8 inflammasome. Nat Immunol (2012) 13(3):246–54. 10.1038/ni.2222 22267217

[116] Zwolanek F Riedelberger M Stolz V Jenull S Istel F Koprulu AD The non-receptor tyrosine kinase Tec controls assembly and activity of the noncanonical caspase-8 inflammasome. PLoS Pathog (2014) 10(12):e1004525. 10.1371/journal.ppat.1004525 25474208 PMC4256681

[117] Chang TH Huang JH Lin HC Chen WY Lee YH Hsu LC Dectin-2 is a primary receptor for NLRP3 inflammasome activation in dendritic cell response to *Histoplasma capsulatum*. PLoS Pathog (2017) 13(7):e1006485. 10.1371/journal.ppat.1006485 28671985 PMC5510910

[118] Tavares AH Magalhaes KG Almeida RD Correa R Burgel PH Bocca AL. NLRP3 inflammasome activation by *Paracoccidioides brasiliensis*. PLoS Negl Trop Dis (2013) 7(12):e2595. 10.1371/journal.pntd.0002595 24340123 PMC3855149

[119] Guo C Chen M Fa Z Lu A Fang W Sun B Acapsular *Cryptococcus neoformans* activates the NLRP3 inflammasome. Microbes Infect (2014) 16(10):845–54. 10.1016/j.micinf.2014.08.013 25193031

[120] Said-Sadier N Padilla E Langsley G Ojcius DM. *Aspergillus fumigatus* stimulates the NLRP3 inflammasome through a pathway requiring ROS production and the Syk tyrosine kinase. PLoS One (2010) 5(4):e10008. 10.1371/journal.pone.0010008 20368800 PMC2848854

[121] Mao L Zhang L Li H Chen W Wang H Wu S Pathogenic fungus *Microsporum canis* activates the NLRP3 inflammasome. Infect Immun (2014) 82(2):882–92. 10.1128/IAI.01097-13 24478101 PMC3911390

[122] Kistowska M Fenini G Jankovic D Feldmeyer L Kerl K Bosshard P *Malassezia* yeasts activate the NLRP3 inflammasome in antigen-presenting cells *via* Syk-kinase signalling. Exp Dermatol (2014) 23(12):884–9. 10.1111/exd.12552 25267545

[123] Joly S Ma N Sadler JJ Soll DR Cassel SL Sutterwala FS. Cutting edge: *Candida albicans* hyphae formation triggers activation of the Nlrp3 inflammasome. J Immunol (2009) 183(6):3578–81. 10.4049/jimmunol.0901323 19684085 PMC2739101

[124] Gow NA van de Veerdonk FL Brown AJ Netea MG. *Candida albicans* morphogenesis and host defence: discriminating invasion from colonization. Nat Rev Microbiol (2011) 10(2):112–22. 10.1038/nrmicro2711 22158429 PMC3624162

[125] Krysan DJ Sutterwala FS Wellington M Catching fire: *Candida albicans*, macrophages, and pyroptosis. PLoS Pathog (2014) 10(6):e1004139 10.1371/journal.ppat.1004139 24967821 PMC4072798

[126] Pietrella D Pandey N Gabrielli E Pericolini E Perito S Kasper L Secreted aspartic proteases of *Candida albicans* activate the NLRP3 inflammasome. Eur J Immunol (2013) 43(3):679–92. 10.1002/eji.201242691 23280543

[127] Karki R Man SM Malireddi RK Gurung P Vogel P Lamkanfi M Concerted activation of the AIM2 and NLRP3 inflammasomes orchestrates host protection against *Aspergillus* infection. Cell Host Microbe (2015) 17(3):357–68. 10.1016/j.chom.2015.01.006 25704009 PMC4359672

[128] Tomalka J Ganesan S Azodi E Patel K Majmudar P Hall BA A novel role for the NLRC4 inflammasome in mucosal defenses against the fungal pathogen *Candida albicans*. PLoS Pathog (2011) 7(12):e1002379. 10.1371/journal.ppat.1002379 22174673 PMC3234225

[129] Rappleye CA Eissenberg LG Goldman WE. *Histoplasma capsulatum* alpha-(1,3)-glucan blocks innate immune recognition by the beta-glucan receptor. Proc Natl Acad Sci U S A (2007) 104(4):1366–70. 10.1073/pnas.0609848104 17227865 PMC1783108

[130] Cao Z Conway KL Heath RJ Rush JS Leshchiner ES Ramirez-Ortiz ZG Ubiquitin ligase TRIM62 regulates CARD9-mediated anti-fungal immunity and intestinal inflammation. Immunity (2015) 43(4):715–26. 10.1016/j.immuni.2015.10.005 26488816 PMC4672733

[131] Xiao Y Tang J Guo H Zhao Y Tang R Ouyang S Targeting CBLB as a potential therapeutic approach for disseminated candidiasis. Nat Med (2016) 22(8):906–14. 10.1038/nm.4141 27428899 PMC4975523

[132] Wirnsberger G Zwolanek F Asaoka T Kozieradzki I Tortola L Wimmer RA Inhibition of CBLB protects from lethal *Candida albicans* sepsis. Nat Med (2016) 22(8):915–23. 10.1038/nm.4134 27428901 PMC6209141

[133] Zhu LL Luo TM Xu X Guo YH Zhao XQ Wang TT E3 ubiquitin ligase Cbl-b negatively regulates C-type lectin receptor-mediated antifungal innate immunity. J Exp Med (2016) 213(8):1555–70. 10.1084/jem.20151932 27432944 PMC4986534

[134] Leach MD Brown AJ. Posttranslational modifications of proteins in the pathobiology of medically relevant fungi. Eukaryot Cell (2012) 11(2):98–108. 10.1128/EC.05238-11 22158711 PMC3272899

[135] Kingeter LM Lin X C-type lectin receptor-induced NF-kappaB activation in innate immune and inflammatory responses. Cell Mol Immunol (2012) 9(2):105–12. 10.1038/cmi.2011.58 22246129 PMC4002809

[136] Gorjestani S Yu M Tang B Zhang D Wang D Lin X Phospholipase Cgamma2 (PLCgamma2) is key component in dectin-2 signaling pathway, mediating anti-fungal innate immune responses. J Biol Chem (2011) 286(51):43651–9. 10.1074/jbc.M111.307389 22041900 PMC3243564

[137] Oykhman P Timm-McCann M Xiang RF Islam A Li SS Stack D Requirement and redundancy of the Src family kinases Fyn and Lyn in perforin-dependent killing of *Cryptococcus neoformans* by NK cells. Infect Immun (2013) 81(10):3912–22. 10.1128/IAI.00533-13 23918783 PMC3811764

[138] Dennehy KM Ferwerda G Faro-Trindade I Pyz E Willment JA Taylor PR Syk kinase is required for collaborative cytokine production induced through dectin-1 and toll-like receptors. Eur J Immunol (2008) 38(2):500–6. 10.1002/Eji.200737741 18200499 PMC2430329

[139] Deng Z Ma S Zhou H Zang A Fang Y Li T Tyrosine phosphatase SHP-2 mediates C-type lectin receptor-induced activation of the kinase Syk and anti-fungal TH17 responses. Nat Immunol (2015) 16(6):642–52. 10.1038/ni.3155 25915733 PMC4439382

[140] Blanco-Menendez N Del Fresno C Fernandes S Calvo E Conde-Garrosa R Kerr WG SHIP-1 couples to the dectin-1 hemITAM and selectively modulates reactive oxygen species production in dendritic cells in response to *Candida albicans*. J Immunol (2015) 195(9):4466–78. 10.4049/jimmunol.1402874 26416276 PMC4641325

[141] Serezani CH Kane S Medeiros AI Cornett AM Kim SH Marques MM PTEN directly activates the actin depolymerization factor cofilin-1 during PGE2-mediated inhibition of phagocytosis of fungi. Sci Signal (2012) 5(210):ra12. 10.1126/scisignal.2002448 22317922 PMC3400468

[142] Liu Q Zhou H Langdon WY Zhang J. E3 ubiquitin ligase Cbl-b in innate and adaptive immunity. Cell Cycle (2014) 13(12):1875–84. 10.4161/cc.29213 24875217 PMC4111751

[143] Liu YC Ubiquitin ligases and the immune response. Annu Rev Immunol (2004) 22:81–127. 10.1146/annurev.immunol.22.012703.104813 15032575

[144] Reyes-Turcu FE Ventii KH Wilkinson KD. Regulation and cellular roles of ubiquitin-specific deubiquitinating enzymes. Annu Rev Biochem (2009) 78:363–97. 10.1146/annurev.biochem.78.082307.091526 19489724 PMC2734102

[145] Zinngrebe J Montinaro A Peltzer N Walczak H. Ubiquitin in the immune system. EMBO Rep (2014) 15(1):28–45. 10.1002/embr.201338025 24375678 PMC4303447

[146] Bhoj VG Chen ZJ. Ubiquitylation in innate and adaptive immunity. Nature (2009) 458(7237):430–7. 10.1038/nature07959 19325622

[147] Huang F Xiao H Sun BL Yang RG. Characterization of TRIM62 as a RING finger E3 ubiquitin ligase and its subcellular localization. Biochem Biophys Res Commun (2013) 432(2):208–13. 10.1016/j.bbrc.2013.02.012 23402750

[148] Zhang J. Ubiquitin ligases in T cell activation and autoimmunity. Clin Immunol (2004) 111(3):234–40. 10.1016/j.clim.2004.02.003 15183144

[149] Shembade N Ma A Harhaj EW. Inhibition of NF-kappaB signaling by A20 through disruption of ubiquitin enzyme complexes. Science (2010) 327(5969):1135–9. 10.1126/science.1182364 20185725 PMC3025292

[150] Skaug B Chen J Du F He J Ma A Chen ZJ. Direct, noncatalytic mechanism of IKK inhibition by A20. Mol Cell (2011) 44(4):559–71. 10.1016/j.molcel.2011.09.015 22099304 PMC3237303

[151] Kanayama M Inoue M Danzaki K Hammer G He YW Shinohara ML Autophagy enhances NFkappaB activity in specific tissue macrophages by sequestering A20 to boost antifungal immunity. Nat Commun (2015) 6:5779 10.1038/ncomms6779 25609235 PMC4304414

